# Resources are associated with functional outcome and brain morphometry in childhood cancer survivors

**DOI:** 10.3389/fonc.2026.1886395

**Published:** 2026-08-06

**Authors:** Saskia Salzmann, Valentin Benzing, David Romascano, Regula Everts

**Affiliations:** 1 Division of Neuropaediatrics, Development and Rehabilitation, Department of Paediatrics, Inselspital, Bern University Hospital, University of Bern, Bern, Switzerland; 2 Graduate School for Health Sciences, University of Bern, Bern, Switzerland; 3 Institute of Sport Science, University of Bern, Bern, Switzerland; 4 Support Center for Advanced Neuroimaging (SCAN), University Institute of Diagnostic and Interventional Neuroradiology, Inselspital, Bern University Hospital and University of Bern, Bern, Switzerland; 5 Division of Paediatric Hematology and Oncology, Department of Paediatrics, Inselspital, Bern University Hospital, University of Bern, Bern, Switzerland

**Keywords:** brain morphometry, childhood cancer, cognition, contextual resources, individual resources, motor functions, multifactorial

## Abstract

**Background:**

Childhood cancer survivors exhibit a high prevalence of long-term cognitive and motor impairments as well as structural brain alterations. Examining late effects from a multimodal perspective is essential as individual and contextual resources (e.g. self-efficacy, optimism; parental support, socioeconomic status) as well as risk factors (e.g. disease and treatment related factors) likely impact neurodevelopment such as functional outcome (cognition, motor function) and brain structure in childhood cancer survivors. It is purely understood how individual and contextual resources are associated with functional outcome and brain structure and whether resources can moderate the relationship between functional outcome and brain structure. A deeper understanding of these interrelations can help to identify components for multimodal interventions to strengthen neurodevelopment after childhood cancer in the long-term. This exploratory study investigates associations between resources (individual and contextual) and neurodevelopmental outcomes and how resources moderate functional outcome, and brain morphometry in childhood cancer survivors.

**Methods:**

56 childhood cancer survivors (N = 45 non central nervous system cancer survivors (non-CNS), N = 11 CNS cancer survivors (CNS)) and 45 typically developing children all aged 7–16 years; M = 10.85, SD = 2.52)) underwent structural MRI, cognitive and motor assessments and filled out questionnaires on individual and contextual resources at least one year after the end of cancer treatment. Correlations were computed, followed by principal components analysis to minimize factors (given the small sample and number of variables), and subsequent moderation analyses were conducted.

**Results:**

More and stronger correlations occurred between resources and functional outcome as well as resources and brain morphometry in survivors (non-CNS: N correlations = 16, r = 0.32-0.61; CNS: N correlation = 9, r = 0.78-0.97) compared with typically developing children (N correlation = 2, r = 0.34-0.37), with the strongest effects in CNS cancer survivors. Interaction effects suggest that resources may moderate the associations between cognition and motor function (β=-0.834-0.894, p_uncorr_=0.003-0.047), as well as between cognition and brain morphometry (β=-1.185 to -1.109, p_uncorr_=0.034-0.046). The associations across resources, cognition, motor function, and brain morphometry were more pronounced in CNS and non-CNS cancer survivors (non-CNS: N = 34, r=0.33-0.61; CNS: N = 29, r=0.72-1.00) compared to typically developing children (N = 17, r=0.31-0.47).

**Conclusion:**

These exploratory findings propose that individual and contextual resources are associated with functional outcome and brain morphometry following childhood cancer, suggesting that resource-oriented, multimodal interventions should be considered for neurodevelopmental rehabilitation.

## 1 Introduction

In the last decades the survival rate after childhood cancer increased up to 80% (1). However, cancer and its treatment can impede neurodevelopment in children and adolescents and often lead to structural alterations in the brain (2–10). Therefore, patients’ long-term outcomes and post-treatment care are becoming increasingly important.

Individual (within the child) and contextual (social & structural) resources (11, 12) play an important role in the development of cognitive and motor functions (13, 14) as well as for structural brain development during childhood and adolescence (15–17). These resources evolve over time and emerge from complex interactions across biological, psychological, and physical characteristics, family and peer relationships, and socioeconomic factors (11, 12, 18). Resources are known to support adaptive functioning and resilience in the context of adversity (5, 6, 19).

Approximately half of childhood cancer survivors demonstrate age-appropriate cognitive functioning, while up to 48% and 70% experience long-term cognitive and motor impairments, respectively (2, 6, 8) and some have structural brain alterations in the long-term (4). This variability stems from an interplay between risk and protective factors such as biological, medical, developmental and psychosocial characteristics. All of these variables interact and shape long-term outcomes following childhood cancer (5, 6). This multifactorial perspective aligns with the diathesis–stress model, which proposes that outcomes emerge from the interaction between underlying vulnerability (diathesis) and contextual or biological stressors, while protective factors may buffer adverse effects and promote resilience (20). Childhood and adolescence are critical periods for brain development in which cancer diagnosis and its treatment can function as significant early-life stressors that can alter neurodevelopmental trajectories. The combined burden of early adversity and cancer-related treatments likely affects brain development, with lasting effects on cognition, behavior, and emotional outcome (21).

Research about the influence of individual and contextual resources on outcome after childhood cancer remains limited. Previous analysis of parts of this study cohort suggests that childhood cancer survivors differed in certain resource domains, such as peer integration and physical self-concept compared to typically developing children (2, 14). Moreover, social resources (e.g. parental support, peer integration) and personal resources (e.g. optimism, self-efficacy) have been associated with cognitive functions (14). More broadly, a link between resources and cognitive and psychosocial outcomes has been suggested also in other studies (22–27). A review on healthy child development concludes that there are significant associations between socioeconomic factors and variations in brain structure, particularly in regions related to memory, executive functioning, and emotion regulation (15).

A large cohort study with adolescents found that life factors (e.g. neighborhood environment, parental characteristics, quality of family life, perinatal history, cardiometabolic health, cognition, and psychopathology) are associated with brain organization in youth. These life factors tend to cluster along a continuum ranging from advantage to adversity rather than occurring in isolation. As a result, examining single factors alone may overlook the broader context shaping brain development (16). In childhood cancer survivors’ late effects in cognitive and motor functions, as well as brain structure have been mostly studied individually (2, 3, 5, 7).

The aim of this exploratory study is to gain a multimodal perspective on neurodevelopmental late-effects (cognition, motor functions, brain morphometry) and to examine how individual and contextual resources are associated with functional outcomes (cognition and motor functions) and brain morphometry (cortical thickness, surface area and subcortical volume) in childhood cancer survivors. Further, we examine the moderating role of resources on the associations between cognition, motor functions and brain morphometry. We hypothize that resources moderate the association between brain morphometry and functional outcome. Children with stronger resources may be better able to compensate for cancer and treatment-related structural brain changes or cognitive and motor impairments. Our study will give insight into how an adverse event such as childhood cancer shapes the interaction between resources and neurodevelopmental outcomes.

## 2 Materials and methods

This a cross-sectional, retrospective analysis of data collected within the Brainfit study (28), which investigated the effects of cognitive and physical training in childhood cancer survivors. For the present study, the data of the first timepoint (before training) was used. Written informed consent was obtained from parents/legal guardians and participants aged ≥14 years. This study was conducted in the cantons of Bern and Zurich, Switzerland, between January 2017 and December 2018. It was granted ethical approval by the respective cantonal ethics committees (Bern: KEK-NR. 196/15; Zurich: ZH2015-03997) and was registered at ClinicalTrials.gov (NCT02749877). The study was conducted in accordance with the current version of the World Medical Association Declaration of Helsinki (Declaration of Helsinki, 2013), and ICH-GCP guidelines as well as the local legally applicable requirements.

### 2.1 Participants

In total 56 children with a history of central nervous (CNS; N = 11) or non-central nervous cancer (non-CNS; N = 45) and 45 typically developing children were included in the analysis. Sample size calculation was performed *a priori* as described in Benzing et al. (2018 (25)).

#### 2.1.1 Childhood cancer survivors

Childhood cancer survivors treated at the University Children’s Hospitals Bern or Zurich were recruited through the Swiss Childhood Cancer Registry of Switzerland. Inclusion criteria were (a) diagnosis of cancer within the past 10 years (2007 to 2017) (b) age between 7 and 16 years at the time of assessment (c) at least one year off-therapy (d) treatment involved chemotherapy and/or radiotherapy, and - among those with CNS tumors - also surgery. Excluded were survivors (a) with surgical removal of the tumor without radiation and/or chemotherapy (only in non-CNS) (b) dental braces, metal parts in the body (c) an insufficient quality in the T1 weighted MR imaging.

#### 2.1.2 Typically developing children (controls)

Typically developing children were recruited through advertising and siblings of the survivors. Inclusion criteria were (a) age between 7 and 16 years old (b) normal or corrected-to-normal vision and hearing. Excluded were children with (a) chronic illness (e.g. birth deformity, congenital heart defect, cerebral palsy, or epilepsy) (b) medical problems potentially influencing development (e.g. meningitis, traumatic brain injury) (c) pervasive development disorders (e.g. autism) (d) metal parts in the body (e) an insufficient quality in the T1 weighted MR imaging.

### 2.2 Outcome measures

#### 2.2.1 Demographics

Baseline characteristics (age, sex) and clinical characteristics (cancer type and treatment) were assessed. The cancer types were scored according to the third international classification of childhood cancer (29).

#### 2.2.2 Resources

Socioeconomic status was assessed through the family affluence scale (30), ranging from zero to nine. Resources were assessed with a self-rating resource questionnaire (FRKJ (31);) focusing on personal (empathy, optimism, coherence, self-control, self-efficacy and self-esteem) and social resources (parental support, authoritative parenting style, peer integration and school integration) and the short version of the Physical Self-Description Questionnaire (PSDQ-S (32);) to assess physical self-concept and aspects of self-esteem (global esteem).

#### 2.2.3 Cognitive and motor assessments

Non-verbal IQ [Test of Nonverbal Intelligence, Fourth Edition, TONI-4; (33)], selective attention [(cancellation: Wechsler Intelligence Scale for Children, WISC-IV; (34)], processing speed [processing speed index including coding and symbol search; WISC-IV; (34)], verbal learning and memory [Atlantis and Atlantis delayed; Kaufman Assessment Battery for Children; K-ABC II; learning and recall were combined to an overall verbal memory score; (35)] were assessed. Executive functions were assessed using inhibition [completion time, color-word interference subtest, condition three; Delis-Kaplan Executive Function System, D-KEFS; (36)], shifting [completion time, color-word interference subtest, condition four; D-KEFS; (36)], and working memory [correct trail scores; Block Recall; Working Memory Test Battery for Children, WMTB-C; (37)]. Parents rated everyday executive functions (behavior regulation index) with the parent version of the behavior rating inventory of executive function [BRIEF; (38)]. Gross motor functions (strength, coordination and flexibility) were assessed with the German Motor Test [DMT; (39)]. Fine motor coordination was assessed by the time taken (seconds) to complete the Grooved Pegboard test (40).

#### 2.2.4 Neuroimaging

##### 2.2.4.1 Image acquisition

Magnetic resonance images were acquired using a 3-Tesla Siemens Magnetom Prisma VE11C scanner (Siemens, Erlangen, Germany) using a 64-channel head coil. Structural imaging was performed with a 3-D T1 magnetization-prepared rapid gradient-echo (MPRAGE) sequence (acquisition time, TA = 4:33 min; repetition time, TR = 1950 ms; echo time, TE = 2.19 ms; slices per slap=176; field of view=256×256; isovoxel resolution=1 mm3).

##### 2.2.4.2 Preprocessing and data analysis

Earlier work in this cohort described regional volumetric alterations in non-CNS survivors using voxel-based morphometry (9). The present study characterizes the brain morphometry with surface-based cortical and subcortical morphometric metrics. To derive global and regional brain morphometry, including cortical thickness, cortical surface area and subcortical volume, a Deep Learning-based Anatomy Segmentation and Cortex Parcellation [DL+DiReCT; (41, 42)] on high-resolution structural T1 weighted image was used. DL+DiReCT is a fully automated and publicly available technique (https://github.com/SCAN-NRAD/DL-DiReCT) that integrates segmentation of tissue classes and cortical parcellations, followed by diffeomorphic registration of the gray-white matter boundary to the pial surface to compute a voxel-wise cortical thickness map (DiReCT). We used the “model v6” option for the Desikan-Killiany atlas (43), which resulted in 68 regions of interest (ROIs; N = 34 for each hemisphere). To enable analyses at a broader anatomical scale, individual ROIs were aggregated into composite regions, and measures were averaged across hemispheres. Cortical thickness and surface area were computed for the frontal, temporal, parietal, and occipital lobes, as well as the cingulate and insular cortices. Subcortical volumes were examined for the basal ganglia, diencephalon, and limbic system and infratentorial volume for the brainstem and cerebellum. In childhood cancer survivors with CNS tumors, regions affected by resection cavities were excluded from the analyses. Resection cavities were identified by visual inspection of the structural MRI scans by SS and DR. To avoid distortion of morphometric estimates, the entire composite region was excluded from the corresponding morphometric analyses. Surgery-related morphological alterations beyond the resection cavity were not explicitly controlled for, as the present analyses were intended to capture the overall pattern of brain morphometric differences between childhood cancer survivors and typically developing controls, including those associated with surgical treatment. This should be considered when interpreting the findings.

### 2.3 Statistical analysis

Statistical analyses were conducted using R Studio (Version 2024.12.1 + 563). ChatGPT (OpenAI, GPT-4.5) was used to support the generation and refinement of R code for data processing and visualization. All R codes were independently verified and executed by the authors to ensure accuracy and validity. Non-parametric tests were used because of the small sample size with an alpha level of .05. Demographics and clinical characteristics were compared by group with a Kruskal-Wallis, Chi² test or Mann-Whitney-U Test. To investigate the association across the four domains (resources, cognition, motor functions, brain morphometry), partial correlations controlled for age and sex were conducted (N = 5 resource, N = 9 cognitive, N = 5 motor, N = 16 brain morphometry variables). Statistically significant correlations are presented in chord diagrams generated in R using the circlize package (44). The alpha values of the correlations were adjusted according to the Benjamini-Hochberg procedure (false discovery rate, FDR) to correct for multiple comparison (45).

Principal component analysis (PCA) was performed to reduce the amount of variables in each domain (resources, cognition, motor function, brain morphometry), and to derive uncorrelated components. Further, this data-driven dimensionality reduction approach minimizes the risk of overfitting, and manage Type I error inflation. The first three components of each PCA were retained for subsequent analyses based on eigenvalues >1 and visual inspection of the scree plot (see **Supplementary Figures 1**–**4**). Following the principal component analysis (PCA), factor scores were standardized as z-values. Composite scores derived from PCA were used in subsequent analyses rather than individual measures. At least 1 standard deviation (-1 SD) below the mean was classified as clinically relevant impairment in cognition and motor functions and 1 SD below or above the group mean was classified as meaningful deviation in brain morphometry.

To explore potential moderation effects, LASSO regression models were fitted for each of the four domains (resources, cognition, motor, brain morphometry). Models included resource variables, measures (either cognition, motor or brain morphometry), group, and their interaction terms up to the three-way interaction (measures × resource × group). Cross-validation identified the optimal penalty parameter. Predictors (interactions) with non-zero coefficients were subsequently entered into regression models to estimate effect sizes and assess statistical significance. The alpha values were adjusted for multiple testing (FDR). The non-CNS and CNS cancer survivors were combined to increase statistical power within the moderation analysis.

## 3 Results

### 3.1 Demographics

Demographics and clinical characteristics are presented in **Tables 1**, **2**. Resources, cognitive and motor functions, as well as voxel-based morphometry of a partially overlapping study sample are described in more detail in Benzing et al. [2021 (2)], Siegwart, et al. [2022 (8, 14)] and Spitzhüttl et al. [2021 (9)].

**Table 1:** Demographics and clinical characteristics of participants by group.

Demographics and clinical characteristics	Controls (N = 45)	Non-CNS (N = 45)	CNS (N = 11)	*Statistics*	*p*
Age at assessment (years)	11.07 (2.81)	10.04 (2.19)	11.82 (2.32)	3.175 (2)	.204
Sex (m/f)	24/21 (53.3% m)	26/19 (57.8% m)	7/4 (63.6% m)	0.44 (2)	.880
Socioeconomic status (SES)	6.80 (1.44)	6.73 (1.35)	6.78 (1.72)	0.13 (2)	.940
Age at diagnosis (years)	-	4.80 (3.15)	6.54 (3.16)	162.5	.081
Time since treatment end	-	3.93 (1.96)	4.36 (2.58)	227.0	.676
Treatment duration (months)	-	18.20 (10.72)	12.36 (15.47)	337.5	.060

Data presented as mean (standard deviation) or incidence (%). Group differences: Kruskal-Wallis test (age at assessment, socioeconomic status), Chi-squared test (sex), Mann-Whitney U Test (Age at diagnosis, time since treatment end, treatment duration). Controls, typically developing children; non-CNS, non central nervous system cancer survivors; CNS, central nervous system cancer survivors.

**Table 2:** Clinical characteristics of childhood cancer survivors.

Clinical characteristics	N (%)
Cancer type (non-CNS cancers)	45
Leukemia	27 (60.00)
Lymphoma	6 (13.33)
Neuroblastoma	1 (2.22)
Renal tumor	5 (11.11)
Soft tissue sarcoma	4 (8.88)
Germ cell tumor	1 (2.22)
Other malignant neoplasms and melanomas	1 (2.22)
Cancer Type (CNS and miscellaneous intracranial and intraspinal neoplasms)	11
Ependymomas and choroid plexus tumor	2 (18.18)
Astrocytomas	5 (45.45)
Intracranial and intraspinal embryonal tumors	1 (9.09)
Intracranial and intraspinal germ cell tumors	3 (27.27)
Treatment	N (%)
Chemotherapy only	25 (44.64)
Surgery only	5 (8.93)
Surgery, radiation therapy	4 (7.14)
Surgery, chemotherapy	13 (23.21)
Surgery, chemotherapy, radiation therapy	9 (16.07)

Data presented as incidence (%). Categorization of tumor type according to the ICCC-3, International Classification of Childhood Cancer (29). non-CNS, non central nervous system cancer survivors; CNS, central nervous system cancer survivors.

### 3.2 Associations between resources, functional outcome, and brain morphometry

**Figure 1:**
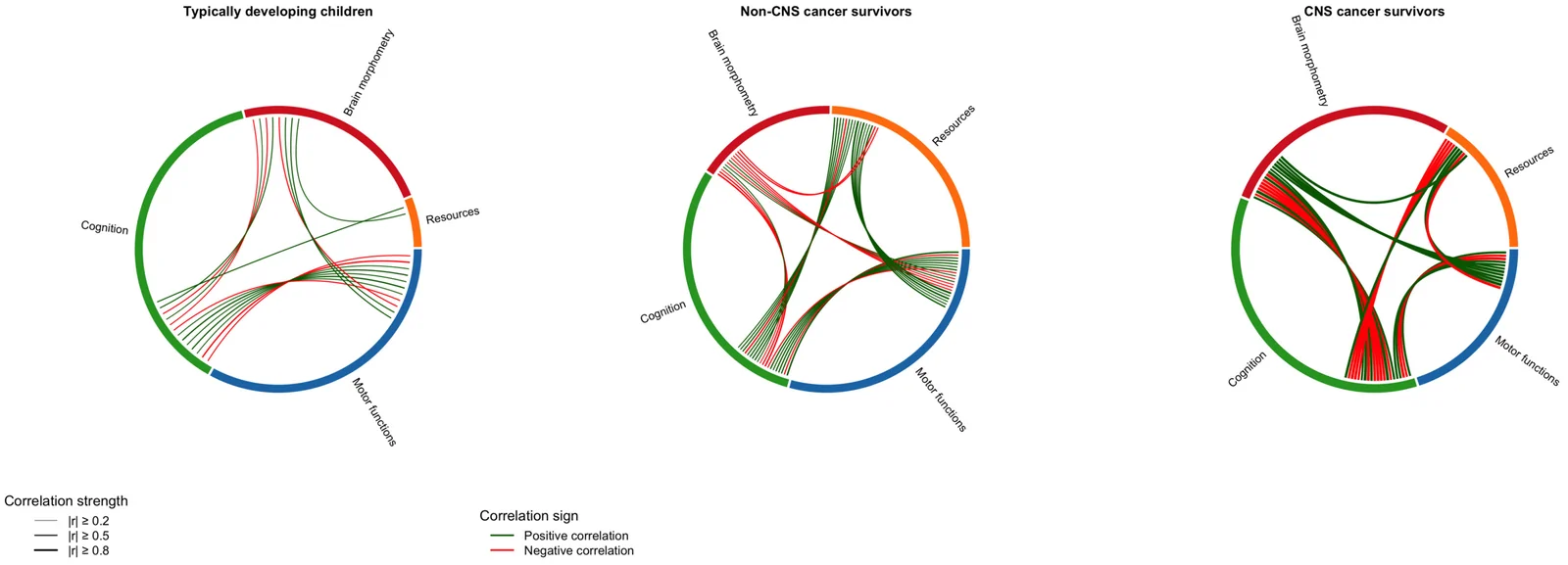
Association pattern across resources, cognition, motor functions and brain morphometry. Negative correlations are shown in red and positive correlations in green, with line thickness representing correlation strength. The extent of the colored blocks in the circular layout corresponds to the number of significant correlations associated with that variable. The chord diagram illustrates significant associations between four domains: resources, cognition, motor functions and brain morphometry. Both non-CNS and CNS cancer survivors show more significant associations across these four domains than typically developing children, with strongest associations observed in CNS cancer survivors. Notably, resources are closer linked to cognition, motor functions and brain morphometry in childhood cancer survivors than in typically developing children.

**Figure 2:**
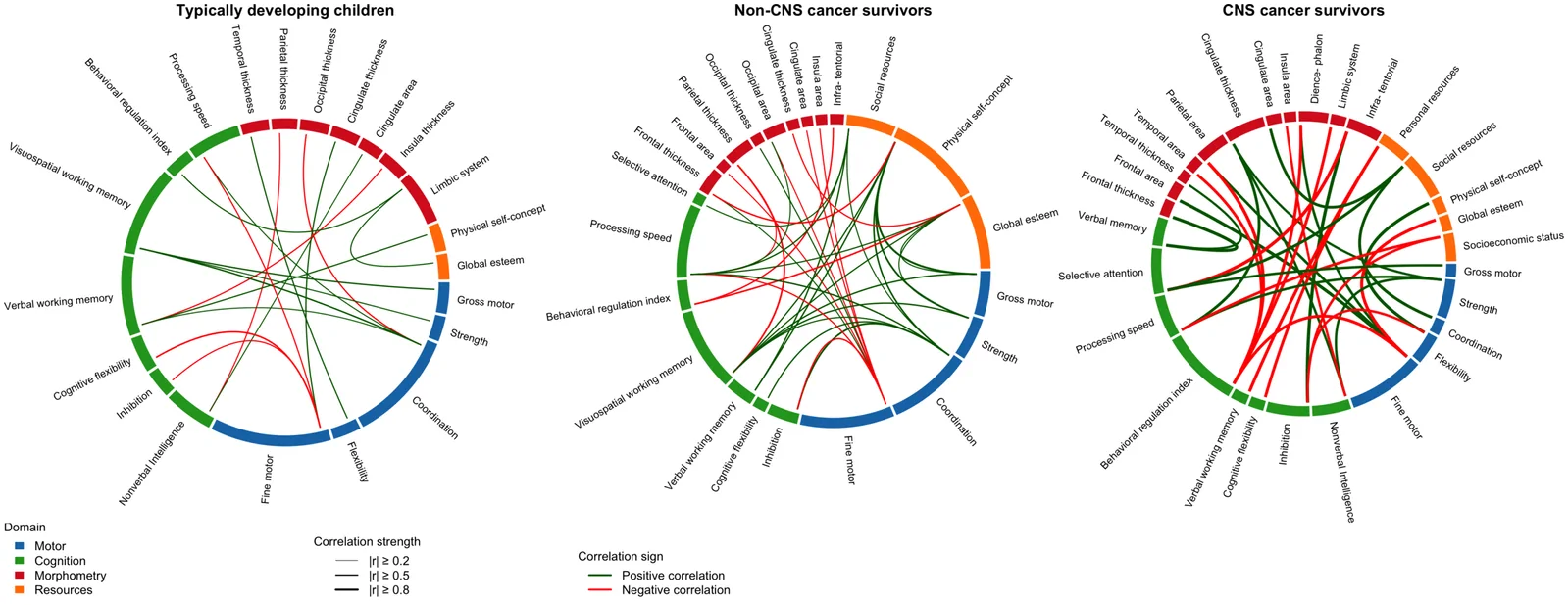
Associations across resources, cognition, motor functions and brain morphometry. Negative correlations are shown in red and positive correlations in green, with line thickness representing correlation strength. The angular position of each variable within the circular layout corresponds to the number of significant correlations associated with that variable. The chord diagram illustrates significant associations between individual variables across four domains: resources, cognition, motor functions, and brain morphometry. Both non-CNS and CNS cancer survivors show more significant associations between individual variables across these four domains than typically developing children, with strongest associations observed in CNS cancer survivors. Notably, resources are closer linked to cognition, motor functions and brain morphometry in childhood cancer survivors than in typically developing children.

In **Figures 1**, **2** significant associations between the four domains resources, cognition, motor functions and brain morphometry are displayed with a chord diagram (for correlations see **Supplementary Table 1**). Pairwise correlations (controlled for age and sex) were computed across all variables to examine the overall association patterns of the four domains, which were subsequently visualized and compared across groups (non-CNS and CNS survivors and controls; **Figures 1**, **2**). In survivors resources were correlated more often (significant correlations: Controls N = 2, non-CNS N = 16, CNS N = 9) and more strongly (Controls: r=0.34-0.37; non-CNS: r=0.32-0.61; CNS: r=0.78-0.97) with cognition, motor functions and brain morphometry than in typically developing children (orange part of circle, see **Figures 1**, **2**). Further, there were more associations across all four domains in non-CNS (N = 34, r=0.33-0.61) and CNS (N = 29, r=0.72-1.00) cancer survivors compared to typically developing children (N = 17, r=0.31-0.47). Across all four domains three correlations within the non-CNS cancer survivors surpassed the correction for multiple comparisons (r=0.52-0.61, pcorr<.001).

### 3.3 Principal component analysis

To reduce the amount of variables included in the correlations across the four domains, principle component analysis was performed. We found three components with eigenvalues >1 in all four domains (for more details about extraction of PCA see **Supplementary Material**). Three PCA loading on clusters within the resource domain were extracted (1) personal and social resources (2), physical self-concept and global esteem and (3) socioeconomic status. For the cognition domain, three PCA appeared (1) verbal and visuospatial working memory and verbal memory (2), inhibition and cognitive flexibility and (3) intelligence, processing speed and attention. For the motor domain we found three PCA (1) strength and coordination (2), finemotor and (3) flexibility. For the brain morphometry domain three PCA were established (1) cortical surface area (2), cortical thickness and (3) subcortical volume.

### 3.4 Clinically significant impairments in functional outcome and alterations in brain morphometry

**Figure 3:**
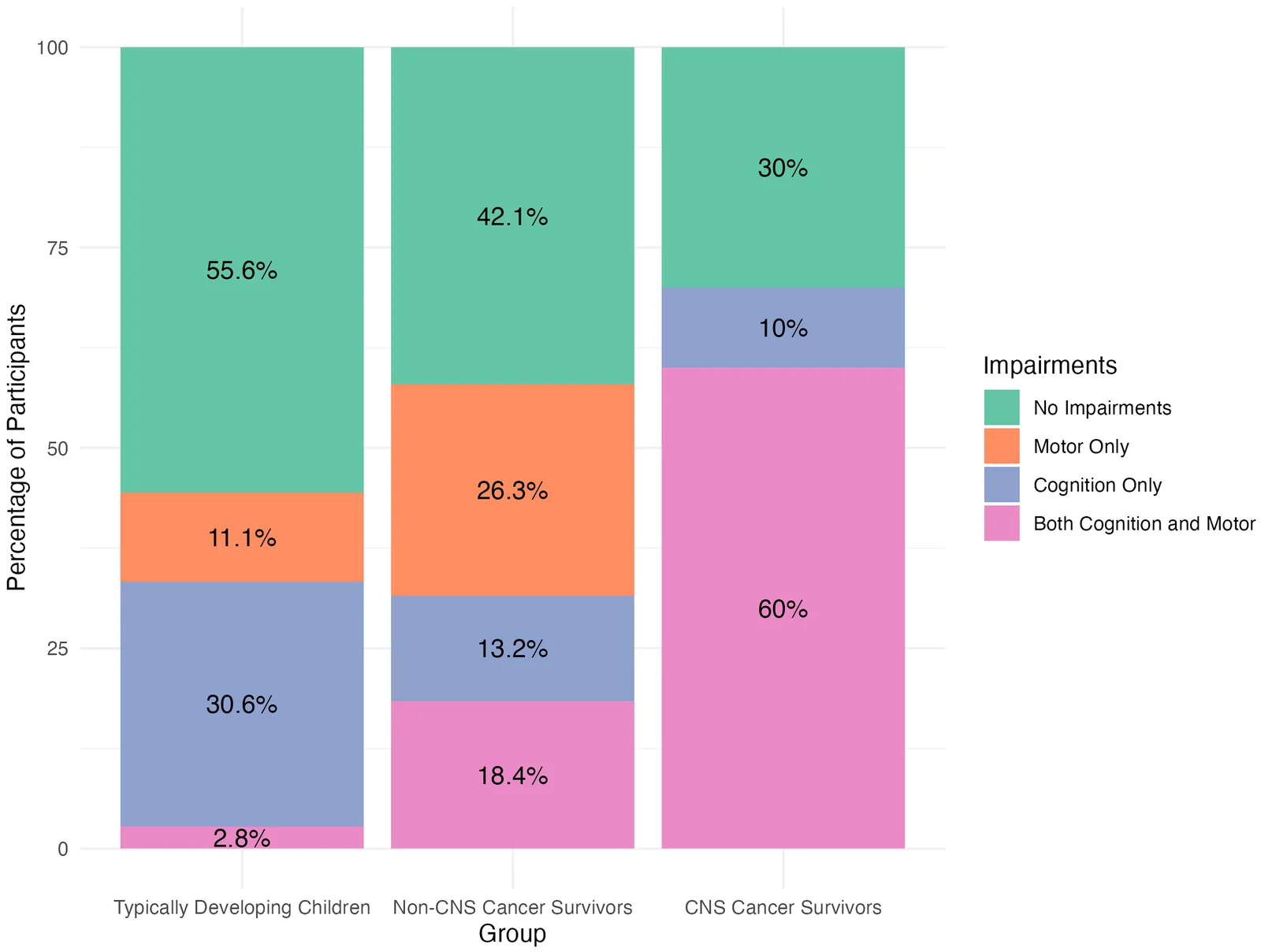
Clinically significant impairments in functional outcome (cognition, motor functions).

In CNS cancer survivors, 30% showed clinically significant impairments (-1 SD) in all three cognitive PCAs (see **Table 3**; **Supplementary Table 2**). In the three motor PCAs 0-54.5% of the CNS cancer survivors showed clinically significant impairments. In non-CNS cancer survivors 7.7-17.9% showed clinically significant impairments in the three cognition PCAs and 9.3-30.2% in the three motor PCAs. In typically developing children, 5.6-16.7% showed clinically significant impairment in cognition PCAs and 4.4-13.3% in the three motor PCAs. Clinically significant impairments in both, the cognitive and motor PCAs occurred in 60% of the CNS cancer survivors, in 18.4% of the non-CNS cancer survivors and in 2.8% of the typically developing children (see **Figure 3**). Brain morphometric deviations in the three PCAs exceeded at least one standard deviation in 15.6-24.4% of non-CNS survivors and in 4.4-37.8% of typically developing children. Both cognitive impairments and brain morphometric deviations occurred in 7.7% of non-CNS cancer survivor and in none of the typically developing children. Both motor impairments and brain morphometric deviations occurred in 7.0% of the non-CNS cancer survivors and 2.2% of the typically developing children.

**Table 3:** Clinically significant impairments (functional outcome) and deviations from the mean (brain morphometry).

Clinically significant impairments (-1.0 SD)	Controls			Non-CNS			CNS		
	N total	N 1SD	%	N total	N 1SD	%	N total	N 1SD	%
Cognition									
PC1 (visual and verbal working memory, verbal memory)	36	5	13.9	39	7	17.9	10	3	30.0
PC2 (inhibition, cognitive flexibility)	36	2	5.6	39	5	12.8	10	3	30.0
PC3 (selective attention, processing speed)	36	6	16.7	39	3	7.7	10	3	30.0
Motor functions									
PC1 (strength, coordination)	45	2	4.4	43	8	18.6	11	6	54.5
PC2 (fine motor)	45	6	13.3	43	4	9.3	11	2	18.2
PC3 (flexibility)	45	4	8.9	43	13	30.2	11	0	0.0
Deviation from group mean (+/- 1 SD).									
Brain morphometry									
PC1 (cortical surface area)	45	2	4.4	45	11	15.6	–	–	–
PC2 (cortical thickness)	45	17	37.8	45	11	24.4	–	–	–
PC3 (subcortical volume)	45	14	31.1	45	8	24.4	–	–	–

Data presented as incidence (%). Clinically significant impairments defined as z<-1.0 standard deviation (SD) and deviation from mean as at least one standard deviation from the group. Controls, typically developing children; non-CNS, childhood cancer survivor without involvement of the central nervous system; CNS, childhood cancer survivor with involvement of the central nervous system. No results are presented for CNS cancer survivors in the brain morphometry PCAs because of a to small N due to exclusion of brain regions.

### 3.5 Moderating role of resources

Using LASSO, we found that resources may modulate the associations between cognition PCAs and motor functions, as well as between cognition and brain morphometry PCAs. For motor function and brain morphometry PCA, all interaction terms were reduced to zero, indicating no evidence that resources play a moderating role between those PCA and other PCAs within cognition, motor functions and brain morphometry.

The following interaction effects show that resources may have a potential moderating effect on the relationship between cognition and motor functions, as well as cognition and brain morphometry. For cognition (PC2; inhibition and cognitive flexibility), several interaction effects reached significance: Motor PC1 × Resources PC3 in survivors (β=0.587, puncorr=0.047), Motor PC2 × Resources PC1 in survivors (β=-0.803, puncorr=0.036), Motor PC3 × Resources PC1 (β=0.515, puncorr=0.003) in the total sample, and Morphometric PC1 × Resources PC1 in survivors (β=-1.185, puncorr=0.034). Further, for cognition (PC3; attention and processing speed), several interaction effects reached significance: Motor PC2 × Resources PC1 in total sample (β=-0.834, puncorr=0.004) and in survivors (β=0.894, puncorr=0.009) and Morphometric PC1 × Resources PC1 in survivors (β=-1.109, puncorr=0.046). None of these effects survived false discovery rate (FDR) correction (see **Supplementary Table 3**).

## 4 Discussion

This exploratory study examined how individual and contextual resources are associated with functional outcomes (cognition and motor functions) and brain morphometry in childhood cancer survivors. Further, the study investigated if resources moderate the associations between cognition, motor functions and brain morphometry. Overall, results indicate that resources are more strongly associated with functional outcomes and brain morphometry in childhood cancer survivors than in typically developing children, with the most pronounced effects observed in CNS cancer survivors. Moreover, exploratory interaction effects suggest that resources may influence the coupling between cognition and motor function as well as between cognition and brain morphometry, particularly after an adverse event such as childhood cancer.

Our results suggest that survivors show more numerous and stronger correlations between resources and functional outcomes, as well as with brain morphometry. The strongest associations across the four domains were found in CNS cancer survivors – a result that should be interpreted with caution, given the small sample size. The finding of more numerous and stronger associations in childhood cancer survivors support other studies that found associations between functional outcome and resources in childhood cancer survivors (22–27). Further, our results extend prior work by Siegwart et al. [2022 (14)], suggesting that personal and social resources were significantly correlated with processing speed and quality of life in partially the same childhood cancer survivors (combined non-CNS and CNS) as investigated in the present study. No such associations emerged in typically developing children (14), suggesting that resources may play a role in shaping outcomes particularly in children most vulnerable to disease and treatment-related stress.

Interestingly, the principal component analysis of resource measures yielded a three-factor solution, differentiating (A) personal and social resources that can been compassed in a psychosocial resource factor, (B) physical self-concept and global self-esteem that can be summarized in a self-related factor (C) and a socioeconomic factor. This pattern suggests that individual and contextual resources are two partially overlapping yet distinguishable constructs. This supports a conceptualization in which psychosocial resources reflect the interplay between personal and social domains, self-related resources capture individuals’ self-evaluations and self-perceptions, and socioeconomic status represents structural resources. This is in line with multidimensional resource frameworks emphasizing the hierarchical organization of protective factors across individual, social, and structural levels (11, 12, 46).

The exploratory interaction effects observed in the present study suggest that resources may modulate the strength of associations between cognitive and motor functioning, as well as between cognition and brain morphometry, however, no causal conclusions can be drawn. From a diathesis–stress perspective (20), childhood cancer and its treatment may act as a major biological stressor that interacts with individual vulnerabilities and protective resources to shape long-term outcomes. In this view, individual and contextual resources may not only be associated with functional outcomes and brain morphometry but also moderate the impact of disease and treatment-related stress on functional outcome and brain morphometry. This is consistent with evidence suggesting that the combined effects of the early adversity of the disease and cancer-related neurotoxicity may alter brain development. Individual and contextual resources are suggested to potentially buffer these effects (21).

Overall, we observed more and stronger associations between the four domains (resources, cognition, motor functions, and brain morphometry) in childhood cancer survivors compared to typically developing children. Further, we found higher rates of co-occurring impairments, particularly in CNS cancer survivors, where 60% showed simultaneous cognitive and motor deficits. This finding supports other studies that found close association between cognition and motor functions in children and adolescents (47, 48). It is assumed that these association arises from shared or overlapping neural pathways and a co-activation of brain regions such as the prefrontal cortex, the cerebellum, and the basal ganglia (49, 50). Further, cognitive and motor impairments are likely related to structural brain alteration, as our findings suggest correlations between brain morphometry and functional outcome in childhood cancer survivors. However, it remains unclear to what extent these structural alterations translate into clinically observable symptoms or functional impairment. Our findings have important clinical implications. First, they suggest that resources may be relevant objectives for intervention in vulnerable populations such as childhood cancer survivors. Importantly, interventions should not be limited to the individual level but explicitly incorporate systemic contexts, including family and school environments. In detail, resource-oriented intervention approaches ideally focus on strengthening social support, enhancing self-efficacy and coping, and on promoting physical activity. Given the interplay between cognitive, motor, and psychosocial domains a unified, multicomponent intervention approach that focuses on these domains simultaneously appears particularly promising. However, CNS cancer survivors appear to show greatest vulnerability, indicating a need for particularly intensive or tailored support in this subgroup to counterbalance the adverse impact of the disease and its treatment.

Several limitations should be considered. First, the cross-sectional design precludes any causal inference, and longitudinal studies are needed to determine whether strengthening resources may attenuate functional decline over time. Second, the relatively small sample size in the CNS cancer survivor group (N = 11), compared to the other two groups (each N = 45), limits statistical power and generalizability. Third, the inclusion of a large number of variables increased model complexity, raising the risk of multiple testing issues and potential alpha inflation, while reducing statistical power to detect small to moderate effects. In addition, the sample was heterogeneous with respect to time since diagnosis, cancer type, and treatment modality, duration, and intensity, which likely introduced further variability. Furthermore, potentially relevant confounding variables including education level, neuropsychiatric comorbidities, and radiotherapy-related factors were not systematically controlled for, as comprehensive data were not available for all participants. Given the small sample size and the heterogeneity of diagnoses and treatment modalities, not all factors potentially influencing functional outcomes and brain morphometric alterations could be accounted for in the present study. Additionally, the present study represents a cross-sectional, retrospective analysis of data collected within a randomized controlled trial. Hence, the choice of outcome measures was constrained by the variables available in the existing dataset and could not be adapted to the specific aims of the present investigation. Finally, the reliance on self-reported measures of resources may be subject to response bias and should be complemented by more objective assessments in future studies.

Future studies should include larger and more homogeneous cohorts to enable investigation of diagnosis- and treatment-specific effects and to further validate the present exploratory findings. Given the sample size and exploratory design of the present study, the use of PCA-derived composite scores is methodologically justified. However, it inevitably involves simplification and may obscure associations between specific variables within each of the four domains (e.g. whether particular resource types drive the observed associations). Future studies with larger samples should therefore additionally examining specific resources, cognitive and motor functions and brain regions to better understand which specific variables are most strongly implicated and how they interact. Further, future studies should additionally include measures to assess adaptive functioning and additional psychological and emotional outcome measures. Bayesian statistical approaches may represent a valuable alternative for future studies with small sample sizes.

### 4.1 Conclusion

In summary, these exploratory findings suggest that individual and contextual resources are more strongly linked to functional outcome and brain morphometry in childhood cancer survivors than in typically developing children, particularly in CNS cancer survivors. Overall, resources may be most impactful after adversity, potentially influencing functional outcomes after childhood cancer. Our results highlight the need for individualized, multimodal interventions, with a focus on individual and contextual resources as a promising complementary approach to reduce cognitive and motor impairments.

## Data Availability

All anonymized data are available from the last author on reasonable request. Requests to access these datasets should be directed to RE, regula.everts@insel.ch.
