# Supplementary material for: Resources are associated with functional outcome and brain morphometry in childhood cancer survivors
Source: Front Oncol. 2026 Aug 6;16:1886395. doi: 10.3389/fonc.2026.1886395 (PMC13489876; doi:10.3389/fonc.2026.1886395)
Supplement: Supplementary file 1 [file DataSheet1.docx]

Supplementary Material

# 1 Supplementary text: principal component analysis

To reduce the number of variables, PCA was performed. PCA of the resource values extracted from all significant clusters revealed 3 components with eigenvalues >1 (see Supplementary Figure 1), explaining 85% of the total variance (principal component 1 = 33%, principal component 2 = 32%, principal component 3 = 20%). Principal component 1 exhibited high loadings on clusters reflecting personal and social resources of the FRKJ, principal component 2 strongly loaded on clusters reflecting physical self-concept and global esteem of the PSDQ-S and principal component 3 on socioeconomic status.

PCA of the cognition domain extracted from all significant clusters revealed 3 components with eigenvalues >1 (see Supplementary Figure 2), explaining 64% of the total variance (principal component 1 = 24%, principal component 2 = 19%, principal component 3 = 21%). Principal component 1 exhibited high loadings on clusters reflecting verbal and visuospatial working memory and verbal memory, with the highest loadings observed in the visuospatial working memory. Principal component 2 strongly loaded on clusters reflecting inhibition and cognitive flexibility and principal component 3 on clusters reflecting intelligence, processing speed and attention.

PCA of the motor values extracted from all significant clusters revealed 3 components with eigenvalues >1 (see Supplementary Figure 3), explaining 92% of the total variance (principal component 1 = 39%, principal component 2 = 28%, principal component 3 = 25%). Principal component 1 exhibited high loadings on clusters reflecting strength and coordination, with the highest loadings observed in strength. Principal component 2 strongly loaded on clusters reflecting finemotor and principal component 3 on flexibility.

PCA of the brain morphometry values extracted from all significant clusters revealed 3 components with eigenvalues >1 (see Supplementary Figure 4), explaining 79% of the total variance (principal component 1 = 33%, principal component 2 = 28%, principal component 3 = 19%). Principal component 1 exhibited high loadings on clusters reflecting cortical surface area values, principal component 2 on clusters reflecting cortical thickness values and principal component 3 on clusters reflecting subcortical volume.

# 2 Supplementary Tables

**Table S1**

Significant correlations between cognition, motor functions, resources and structural brain metrics in typically developing children (controls), non-CNS and CNS cancer survivors

|  |  | Controls | |  | Non-CNS | |  | CNS | |  |
| --- | --- | --- | --- | --- | --- | --- | --- | --- | --- | --- |
|  |  | *r* | *p* | *N* | *r* | *p* | *N* | *r* | *p* | *N* |
| **(a) Cognition** | **Motor** |  |  |  |  |  |  |  |  |  |
| Non-verbal intelligence | Strength |  |  |  |  |  |  | 0.76 | 0.018 | 11 |
| Inhibition | Coordination |  |  |  | 0.53 | 0.001 | 39 |  |  |  |
|  | Flexibility |  |  |  |  |  |  | -0.74 | 0.023 | 11 |
|  | Fine motor | 0.36 | 0.025 | 40 | 0.42 | 0.009 | 40 |  |  |  |
| Cognitive flexibility | Coordination |  |  |  | 0.41 | 0.013 | 39 |  |  |  |
|  | Fine motor | 0.47 | 0.003 | 40 |  |  |  |  |  |  |
| Verbal working memory | Coordination | 0.31 | 0.041 | 45 |  |  |  |  |  |  |
| Visuospatial working memory | Gross motor | 0.41 | 0.009 | 42 | 0.43 | 0.006 | 43 |  |  |  |
|  | Strength | 0.33 | 0.038 | 42 | 0.42 | 0.006 | 43 |  |  |  |
|  | Coordination | 0.44 | 0.005 | 42 | 0.36 | 0.023 | 43 |  |  |  |
| Behavioral regulation index | Fine motor |  |  |  |  |  |  | -0.91 | 0.011 | 8 |
| Processing speed | Strength |  |  |  |  |  |  | 0.78 | 0.014 | 11 |
|  | Coordination | 0.32 | 0.034 | 45 | 0.44 | 0.004 | 43 |  |  |  |
|  | Fine motor | 0.37 | 0.016 | 45 | 0.39 | 0.012 | 43 |  |  |  |
| Selective attention | Gross motor |  |  |  |  |  |  | 0.77 | 0.016 | 11 |
| **(b) Cognition** | **Structural brain metrics** |  |  |  |  |  |  |  |  |  |
| Non-verbal intelligence | Parietal thickness | -0.34 | 0.027 | 45 |  |  |  |  |  |  |
|  | Cingulate thickness |  |  |  |  |  |  | 0.76 | 0.027 | 10 |
|  | Cingulate surface area | 0.31 | 0.044 | 45 |  |  |  |  |  |  |
|  | Insula surface area |  |  |  |  |  |  | -0.73 | 0.039 | 10 |
| Inhibition | Infratentorial volume |  |  |  |  |  |  | 1.00 | 0.018 | 5 |
| Verbal working memory | Insula thickness | -0.37 | 0.015 | 45 |  |  |  |  |  |  |
| Visuospatial working memory | Parietal thickness |  |  |  | -0.52 | <.001* | 44 |  |  |  |
| Behavioral regulation index | Temporal surface area |  |  |  |  |  |  | 0.86 | 0.026 | 8 |
|  | Parietal surface area |  |  |  |  |  |  | 0.98 | 0.004 | 7 |
|  | Diencephalon volume |  |  |  |  |  |  | 0.92 | 0.008 | 8 |
|  | Limbic system volume | -0.36 | 0.019 | 43 |  |  |  | 0.90 | 0.013 | 8 |
|  | Infratentorial volume |  |  |  | 0.43 | 0.005 | 43 |  |  |  |
| Processing speed | Parietal thickness |  |  |  | -0.31 | 0.044 | 44 |  |  |  |
|  | Occipital surface area |  |  |  | 0.33 | 0.035 | 44 |  |  |  |
|  | Cingulate thickness |  |  |  |  |  |  | 0.87 | 0.005 | 10 |
| Selective attention | Infratentorial volume |  |  |  |  |  |  | -1.00 | 0.045 | 5 |
| Verbal memory | Frontal thickness |  |  |  |  |  |  | 0.97 | 0.035 | 6 |
|  | Temporal thickness |  |  |  |  |  |  | 0.68 | 0.045 | 11 |
| **(c) Cognition** | **Resources** |  |  |  |  |  |  |  |  |  |
| Inhibition | Socioeconomis status |  |  |  |  |  |  | -0.78 | 0.040 | 9 |
| Cognitive flexibility | Personal resources |  |  |  |  |  |  | -0.90 | 0.038 | 7 |
| Verbal working memory | Personal resources |  |  |  |  |  |  | -0.94 | 0.018 | 7 |
|  | Social resources |  |  |  | 0.42 | 0.011 | 37 |  |  |  |
|  | Physical self-concept | 0.37 | 0.027 | 38 | 0.43 | 0.008 | 39 |  |  |  |
| Visuospatial working memory | Physical self-concept |  |  |  | 0.40 | 0.013 | 39 |  |  |  |
|  | Global esteem |  |  |  | 0.38 | 0.019 | 40 |  |  |  |
| Behavioral regulation index | Global esteem |  |  |  | 0.48 | 0.002 | 40 |  |  |  |
| Processing speed | Social resources |  |  |  | 0.40 | 0.017 | 37 |  |  |  |
|  | Global esteem |  |  |  | 0.37 | 0.022 | 40 |  |  |  |
|  | Socioeconomis status |  |  |  |  |  |  | -0.84 | 0.018 | 9 |
| Selective attention | Social resources |  |  |  | 0.34 | 0.043 | 37 | 0.89 | 0.041 | 7 |
| **(d) Motor** | **Structural brain metrics** |  |  |  |  |  |  |  |  |  |
| Strength | Cingulate thickness |  |  |  |  |  |  | 0.72 | 0.045 | 10 |
| Coordination | Occipital thickness | -0.39 | 0.009 | 45 |  |  |  |  |  |  |
| Flexibility | Temporal thickness | 0.37 | 0.014 | 45 |  |  |  |  |  |  |
| Fine motor | Frontal thickness |  |  |  | -0.32 | 0.039 | 44 |  |  |  |
|  | Frontal surface area |  |  |  | 0.34 | 0.029 | 44 | -0.97 | 0.027 | 6 |
|  | Parietal surface area |  |  |  |  |  |  | -0.82 | 0.014 | 10 |
|  | Occipital thickness |  |  |  | -0.35 | 0.023 | 44 |  |  |  |
|  | Occipital surface area |  |  |  | 0.33 | 0.032 | 44 |  |  |  |
|  | Cingulate thickness | -0.38 | 0.011 | 45 |  |  |  |  |  |  |
|  | Cingulate surface area |  |  |  | 0.35 | 0.023 | 44 |  |  |  |
|  | Insula surface area |  |  |  | 0.36 | 0.020 | 44 |  |  |  |
|  | Diencephalon volume |  |  |  |  |  |  | -0.80 | 0.029 | 9 |
| **(e) Motor** | **Resources** |  |  |  |  |  |  |  |  |  |
| Gross motor | Physical self-concept |  |  |  | 0.61 | <.001* | 39 |  |  |  |
|  | Global esteem |  |  |  | 0.34 | 0.039 | 40 |  |  |  |
| Strength | Physical self-concept |  |  |  | 0.55 | <.001* | 39 |  |  |  |
|  | Global esteem |  |  |  | 0.33 | 0.042 | 40 |  |  |  |
| Coordination | Social resources |  |  |  | 0.34 | 0.044 | 37 | 0.90 | 0.040 | 7 |
|  | Physical self-concept |  |  |  | 0.48 | 0.002 | 39 |  |  |  |
| Flexibility | Physical self-concept |  |  |  |  |  |  | 0.97 | 0.032 | 6 |
| Fine motor | Global esteem |  |  |  |  |  |  | 0.95 | 0.012 | 7 |
| **(f) Resources** | **Structural brain metrics** |  |  |  |  |  |  |  |  |  |
| Social resources | Cingulate surface area |  |  |  |  |  |  |  |  |  |
| Physical self-concept | Frontal thickness |  |  |  | -0.44 | 0.006 | 40 |  |  |  |
| Global esteem | Cingulate thickness |  |  |  | -0.38 | 0.018 | 41 |  |  |  |
|  | Limbic system volume | 0.34 | 0.041 | 39 |  |  |  | 0.88 | 0.048 | 7 |

*Note.* Controls = typically developing children; non-CNS = childhood cancer survivor without involvement of the central nervous system; CNS = childhood cancer survivor with involvement of the central nervous system; r = partial correlation coefficient (small effect r = 0.10, medium effect r = 0.30, large effect r = 0.50); p = significance (Significant after FDR correction = *).

**Table S2**

Mean values and standard deviations of resource, cognition, motor and morphometric principal components in typically developing children (controls), non-CNS and CNS cancer survivors

|  | **Controls** | **Non-CNS** | **CNS** |
| --- | --- | --- | --- |
| **Resources** |  |  |  |
| PC1 (social and personal resources FRKJ) | 0.06 (0.99) | -0.03 (0.99) | -0.12 (1.28) |
| PC2 (physical self-concept and global esteem PSDQ-S) | 0.20 (1.01) | -0.24 (0.99) | 0.38 (0.73) |
| PC3 (SES) | 0.08 (1.01) | 0.02 (0.96) | -0.59 (1.12) |
| **Cognition** |  |  |  |
| PC1 (visual and verbal working memory, verbal memory) | 0.35 (1.08) | -0.16 (0.86) | -0.63 (0.83) |
| PC2 (inhibition, cognitive flexibility) | 0.20 (1.07) | 0.00 (0.89) | -0.70 (0.92) |
| PC3 (selective attention, processing speed) | -0.05 (1.02) | 0.14 (0.91) | -0.37 (1.25) |
| **Motor functions** |  |  |  |
| PC1 (strength, coordination) | 0.44 (0.86) | -0.20 (0.90) | -1.04 (0.96) |
| PC2 (fine motor) | -0.15 (0.90) | 0.17 (1.11) | -0.07 (0.91) |
| PC3 (flexibility) | 0.12 (1.03) | -0.21 (1.00) | 0.33 (0.72) |
| **Brain morphometry** |  |  |  |
| PC1 (cortical surface area) | 0.06 (0.49) | -0.05 (1.34) | - |
| PC2 (cortical thickness) | 0.01 (1.12) | 0.00 (0.88) | - |
| PC3 (subcortical volume) | 0.22 (0.92) | -0.17 (1.00) | - |

*Notes.* Data presented as mean z-score (standard deviation). Controls = typically developing children; non-CNS = childhood cancer survivor without involvement of the central nervous system; CNS = childhood cancer survivor with involvement of the central nervous system; FRKJ = questionnaire on resources in children and adolescents; PSDQ-S = short version of the Physical Self-Description Questionnaire; SES = Socioeconomic status. No results are presented for CNS cancer survivors in the brain morphometry PCs because of a to small N due to exclusion of brain regions.

**Table S3**

Moderating role of resources in association between cognition and motor functions/structural brain metrics in typically developing children (controls), non-CNS and CNS cancer survivors

| Moderation analysis | Estimate | Std. Error | *Statistic* | *p* | *p FDR adjusted* |
| --- | --- | --- | --- | --- | --- |
| Working memory & memory (Cognition PC1) |  |  |  |  |  |
| Motor PC1 & Resources PC1 | 0.225 | 0.242 | 0.931 | 0.352 | 0.667 |
| Motor PC1 & Resources PC1 (patients) | -0.014 | 0.292 | -0.047 | 0.962 | 0.981 |
| Motor PC1 & Resources PC2 | -0.141 | 0.402 | -0.351 | 0.726 | 0.885 |
| Motor PC1 & Resources PC2 (patients) | 0.509 | 0.437 | 1.163 | 0.245 | 0.595 |
| Motor PC1 & Resources PC3 | 0.038 | 0.299 | 0.126 | 0.900 | 0.949 |
| Motor PC1 & Resources PC3 (patients) | -0.107 | 0.356 | -0.302 | 0.763 | 0.892 |
| Motor PC2 & Resources PC1 | 0.488 | 0.354 | 1.378 | 0.168 | 0.517 |
| Motor PC2 & Resources PC1 (patients) | -0.415 | 0.415 | -0.999 | 0.318 | 0.642 |
| Motor PC3 & Resources PC1 | 0.043 | 0.201 | 0.215 | 0.829 | 0.913 |
| Motor PC3 & Resources PC1 (patients) | 0.248 | 0.253 | 0.981 | 0.327 | 0.643 |
| Morphometric PC1 & Resources PC1 | -0.477 | 0.490 | -0.973 | 0.330 | 0.643 |
| Morphometric PC1 & Resources PC1 (patients) | 0.024 | 0.603 | 0.039 | 0.969 | 0.981 |
| Morphometric PC1 & Resources PC2 | 0.644 | 0.622 | 1.035 | 0.300 | 0.640 |
| Morphometric PC1 & Resources PC2 (patients) | -0.262 | 0.678 | -0.386 | 0.699 | 0.868 |
| Morphometric PC2 & Resources PC1 | -0.137 | 0.234 | -0.585 | 0.559 | 0.821 |
| Morphometric PC2 & Resources PC1 (patients) | 0.178 | 0.341 | 0.521 | 0.602 | 0.830 |
| Morphometric PC3 & Resources PC1 | 0.277 | 0.280 | 0.990 | 0.322 | 0.642 |
| Morphometric PC3 & Resources PC1 (patients) | -0.233 | 0.361 | -0.644 | 0.520 | 0.803 |
| Morphometric PC3 & Resources PC3 | 0.009 | 0.239 | 0.037 | 0.970 | 0.981 |
| Morphometric PC3 & Resources PC3 (patients) | -0.240 | 0.336 | -0.714 | 0.476 | 0.778 |
| Inhibition and Cognitive flexibility (Cognition PC2) |  |  |  |  |  |
| Motor PC1 & Resources PC1 | -0.335 | 0.226 | -1.481 | 0.139 | 0.489 |
| Motor PC1 & Resources PC1 (patients) | 0.523 | 0.274 | 1.911 | 0.056 | 0.429 |
| Motor PC1 & Resources PC2 | -0.410 | 0.356 | -1.152 | 0.249 | 0.595 |
| Motor PC1 & Resources PC2 (patients) | 0.208 | 0.388 | 0.535 | 0.592 | 0.830 |
| Motor PC1 & Resources PC3 | -0.366 | 0.248 | -1.475 | 0.140 | 0.490 |
| Motor PC1 & Resources PC3 (patients) | 0.587 | 0.295 | 1.989 | 0.047* | 0.429 |
| Motor PC2 & Resources PC1 | 0.504 | 0.327 | 1.544 | 0.123 | 0.479 |
| Motor PC2 & Resources PC1 (patients) | -0.803 | 0.383 | -2.096 | 0.036* | 0.406 |
| Motor PC2 & Resources PC2 | 0.678 | 0.490 | 1.384 | 0.166 | 0.517 |
| Motor PC2 & Resources PC2 (patients) | -0.598 | 0.542 | -1.104 | 0.270 | 0.618 |
| Motor PC2 & Resources PC3 | -0.371 | 0.254 | -1.463 | 0.143 | 0.492 |
| Motor PC2 & Resources PC3 (patients) | 0.094 | 0.288 | 0.324 | 0.746 | 0.892 |
| Motor PC3 & Resources PC1 | 0.515 | 0.171 | 3.021 | 0.003* | 0.339 |
| Motor PC3 & Resources PC1 (patients) | -0.275 | 0.215 | -1.281 | 0.200 | 0.540 |
| Morphometric PC1 & Resources PC1 | 0.842 | 0.454 | 1.853 | 0.064 | 0.429 |
| Morphometric PC1 & Resources PC1 (patients) | -1.185 | 0.558 | -2.123 | 0.034* | 0.406 |
| Morphometric PC1 & Resources PC2 | -0.177 | 0.542 | -0.326 | 0.744 | 0.892 |
| Morphometric PC1 & Resources PC2 (patients) | 0.347 | 0.590 | 0.587 | 0.557 | 0.821 |
| Morphometric PC2 & Resources PC1 | 0.188 | 0.190 | 0.994 | 0.320 | 0.642 |
| Morphometric PC2 & Resources PC1 (patients) | 0.078 | 0.276 | 0.284 | 0.776 | 0.892 |
| Morphometric PC3 & Resources PC1 | -0.090 | 0.217 | -0.413 | 0.680 | 0.860 |
| Morphometric PC3 & Resources PC1 (patients) | 0.034 | 0.281 | 0.123 | 0.902 | 0.949 |
| Morphometric PC3 & Resources PC2 | 0.238 | 0.234 | 1.018 | 0.309 | 0.642 |
| Morphometric PC3 & Resources PC2 (patients) | -0.032 | 0.265 | -0.120 | 0.904 | 0.949 |
| Morphometric PC3 & Resources PC3 | -0.107 | 0.190 | -0.564 | 0.573 | 0.824 |
| Morphometric PC3 & Resources PC3 (patients) | -0.119 | 0.267 | -0.446 | 0.656 | 0.856 |
| Attention and processing speed |  |  |  |  |  |
| Motor PC1 & Resources PC1 | -0.320 | 0.239 | -1.339 | 0.181 | 0.524 |
| Motor PC1 & Resources PC1 (patients) | 0.122 | 0.289 | 0.423 | 0.672 | 0.860 |
| Motor PC1 & Resources PC2 | 0.124 | 0.392 | 0.316 | 0.752 | 0.892 |
| Motor PC1 & Resources PC2 (patients) | -0.085 | 0.427 | -0.198 | 0.843 | 0.914 |
| Motor PC1 & Resources PC3 | 0.032 | 0.275 | 0.117 | 0.907 | 0.949 |
| Motor PC1 & Resources PC3 (patients) | -0.231 | 0.327 | -0.706 | 0.480 | 0.778 |
| Motor PC2 & Resources PC1 | -0.834 | 0.293 | -2.843 | 0.004* | 0.339 |
| Motor PC2 & Resources PC1 (patients) | 0.894 | 0.344 | 2.598 | 0.009* | 0.339 |
| Motor PC2 & Resources PC2 | 0.308 | 0.471 | 0.654 | 0.513 | 0.799 |
| Motor PC2 & Resources PC2 (patients) | -0.327 | 0.521 | -0.627 | 0.531 | 0.813 |
| Motor PC2 & Resources PC3 | -0.118 | 0.277 | -0.424 | 0.672 | 0.860 |
| Motor PC2 & Resources PC3 (patients) | 0.130 | 0.315 | 0.412 | 0.680 | 0.860 |
| Motor PC3 & Resources PC1 | 0.118 | 0.194 | 0.606 | 0.545 | 0.820 |
| Motor PC3 & Resources PC1 (patients) | -0.231 | 0.245 | -0.944 | 0.345 | 0.658 |
| Morphometric PC1 & Resources PC1 | 0.866 | 0.451 | 1.918 | 0.055 | 0.429 |
| Morphometric PC1 & Resources PC1 (patients) | -1.109 | 0.555 | -1.999 | 0.046* | 0.429 |
| Morphometric PC1 & Resources PC2 | -0.309 | 0.560 | -0.552 | 0.581 | 0.824 |
| Morphometric PC1 & Resources PC2 (patients) | 0.619 | 0.610 | 1.014 | 0.311 | 0.642 |
| Morphometric PC2 & Resources PC1 | -0.142 | 0.199 | -0.715 | 0.475 | 0.778 |
| Morphometric PC2 & Resources PC1 (patients) | 0.101 | 0.289 | 0.347 | 0.728 | 0.885 |
| Morphometric PC3 & Resources PC1 | -0.227 | 0.254 | -0.894 | 0.371 | 0.681 |
| Morphometric PC3 & Resources PC1 (patients) | 0.066 | 0.328 | 0.200 | 0.841 | 0.914 |
| Morphometric PC3 & Resources PC2 | 0.006 | 0.269 | 0.023 | 0.982 | 0.985 |
| Morphometric PC3 & Resources PC2 (patients) | 0.144 | 0.305 | 0.473 | 0.636 | 0.839 |
| Morphometric PC3 & Resources PC3 | 0.166 | 0.225 | 0.737 | 0.461 | 0.775 |
| Morphometric PC3 & Resources PC3 (patients) | -0.218 | 0.316 | -0.692 | 0.489 | 0.784 |

*Notes.* Results of robust regression analyses predicting Cognition PC1, PC2 and PC3. β = standardized regression coefficients, SE = standard errors, p = significance (α < .05 = *, α < .01 = **). Interaction terms represent moderation effects. All analyses were conducted using a robust regression approach to reduce the influence of outliers.

# 3 Supplementary Figures

**Supplementary Figure 1.** Scree plot showing eigenvalues for each principal component of the resource values. The decision to retain the first three principal components was based on the Kaiser criterion (eigenvalues > 1) and visual inspection of the elbow of the plot.

**Supplementary Figure 2.** Scree plot showing eigenvalues for each principal component of the cognitive values. The decision to retain the first three principal components was based on the Kaiser criterion (eigenvalues > 1) and visual inspection of the elbow of the plot.

**Supplementary Figure 3.** Scree plot showing eigenvalues for each principal component of the motor values. The decision to retain the first three principal components was based on the Kaiser criterion (eigenvalues > 1) and visual inspection of the elbow of the plot.

**Supplementary Figure 4.** Scree plot showing eigenvalues for each principal component of the structural brain metrics. The decision to retain the first three principal components was based on the Kaiser criterion (eigenvalues > 1) and visual inspection of the elbow of the plot.
